# An IoT-Based Computational Framework for Healthcare Monitoring in Mobile Environments

**DOI:** 10.3390/s17102302

**Published:** 2017-10-10

**Authors:** Higinio Mora, David Gil, Rafael Muñoz Terol, Jorge Azorín, Julian Szymanski

**Affiliations:** 1 Department of Computer Science Technology and Computation, University of Alicante, 03690 Alicante, Spain; david.gil@ua.es (D.G.); jazorin@dtic.ua.es (J.A.); 2 Department of Software and Computing Systems, University of Alicante, 03690 Alicante, Spain; rafamt@dlsi.ua.es; 3 Department of Computer Systems Architecture, Gdansk University of Technology, 80-233 Gdansk, Poland; julian.szymanski@eti.pg.gda.pl

**Keywords:** Internet of Things, healthcare monitoring, wearable sensing, sensor network, case studies

## Abstract

The new Internet of Things paradigm allows for small devices with sensing, processing and communication capabilities to be designed, which enable the development of sensors, embedded devices and other ‘things’ ready to understand the environment. In this paper, a distributed framework based on the internet of things paradigm is proposed for monitoring human biomedical signals in activities involving physical exertion. The main advantages and novelties of the proposed system is the flexibility in computing the health application by using resources from available devices inside the body area network of the user. This proposed framework can be applied to other mobile environments, especially those where intensive data acquisition and high processing needs take place. Finally, we present a case study in order to validate our proposal that consists in monitoring footballers’ heart rates during a football match. The real-time data acquired by these devices presents a clear social objective of being able to predict not only situations of sudden death but also possible injuries.

## 1. Introduction

The development of Information and Communications Technologies (ICT) is revolutionizing our lives. They have a great impact on economy [1], policy [2] and other areas of the society. In this way, healthcare has been generally highly connected to technology. This relationship has become stronger over the last two decades. One of the main reasons for this is the proliferation of all types of devices that can be easily installed in most health centers. In addition, telemedicine, which was first mentioned several decades ago, is now a reality and has been highly developed and this evolution has also extended to other healthcare sectors [3].

The emerging paradigm of Internet of Things (IoT) is specially focused on collecting and processing data everywhere and all the time [4,5,6,7]. This expansion of the IoT is allowing to use all type of objects beyond their basic functions, or at least, for those they were designed for a wide range of applications. For example, energy management [8] or analysis of accessibility in smart cities [9]. In the area of healthcare, devices are being designed for many purposes such as patient monitoring to help them manage particularly chronic conditions [10], recovering from injuries [11] or design of Ambient Assisted Living (AAL) environments [12]. Related with this idea, mobile health things (m-health) is a new concept of using smart mobile devices to create efficient healthcare services and solutions [13]. In recent years, a lot of gadgets and smart devices that use sensors to collect information from different parts of the human body have been developed [14]. The devices able to sense human bio-signals are known as biomedical sensors. Their design has been guided by the need to make them lighter, less intrusive for human activity as well as able to provide value-added services to the user. These design criteria have helped to integrate them into the new wearables devices and to popularise them among citizens [15,16]. Moreover, mobile devices are evolved enough and now, these devices are able to work with biomedical data and run health-related Apps [17,18]. In this way, recent applications have been developed around the aforementioned concepts where the sensing capabilities of the ‘things’ play an important role for analysing human behaviour and health [19,20]. There exist many applications and devices that play an increasingly important role in collecting personal health data. Their ability to acquire the electrocardiogram (ECG) data by means of wearable devices, and combine them with users’ mobile devices has enabled the monitoring of heart health metrics for different purposes such as medical care, training and wellness monitoring.

However, sensors and other ‘things’ should have the ability to understand, at least partly, the physical world by themselves [21,22]. Current trends in cognitive science are still a challenge as they require a huge degree of interdisciplinarity. With changes to models of society and recent technologies used in telemedicine as well as in all healthcare sectors, the traditional methods seem insufficient for this new situation. This new state requires using new techniques to address the problem. Although, traditional methods are still valuable, they need to be used together, providing new architecture models.

The focus of the research conducted in this work lies in using these technologies for sensing, and data processing in order to create some intelligence in the devices themselves and collaborate in providing advanced applications to the user. This paper also presents a prototype to demonstrate and validate the proposed solution. The evaluation of the proposal is performed through the assessment of the global performance of the system in delivering computation power. An application example to prevent the health risks associated with physical activity is also described. So, if a medical expert system could detect these causes in real-time during the sports competition by means of analyzing the bio-signals acquired by wearable sensors, medical staff could manage the situation and try to prevent the health risks in sport. As dramatic example of these risks, the sudden arrhythmic death syndrome was the most prevalent cause of Sudden Death (SD) in athletes [23]. Real-time monitoring and analysis of the ECG has proven to be an effective strategy to detect the main abnormalities in the frame of professional sport practice [24].

There are challenges to implementing this idea effectively. The simultaneous monitoring of many athletes could overflow the computing capabilities of the wearables and mobile devices of the in-situ medical staff. This obstacle could be overcome by the deployment of a centralized system that performs a deeper analysis and takes advantage of big data methods on historical aggregated information, although some bottlenecks and delays may result from the communications among the wearable sensors and the centralized expert system.

In this work, a distributed sensing and monitoring framework for Internet of Things’ environments has been proposed to overcome the previous problems. The **aim of this proposal** is to optimize the use of biomedical sensors and computing resources for being able to provide advanced applications to the user. The main contribution of this paper is the design of a distributed computational framework to use the available computing capabilities of the smart devices for sharing the processing of advanced health monitoring applications. The target audience of this work covers an interdisciplinary set of professionals including computer scientists, doctors, coaches, directors of sports centers, among others.

The remaining part of the paper is organized as follows: Section 2 summarizes related literature on the use of wearable devices; Section 3 describes the proposed framework; Section 4 presents the case study of this approach in order to verify the feasibility of our proposal; Finally, Section 5 describes the conclusions and future lines of this research.

## 2. Related Work

There are many issues involved in this work, in which an intensive research activity takes place. This review is focused on the healthcare monitoring area to appreciate the scale of the problem and the recent approaches to face it. Only some of the recent and representative works are presented in order to show the intensiveness and variety of research developed in this area. A final subsection is added, which summarizes the contributions to this work.

### 2.1. Biomedical Sensing and Health Monitoring

One of the prime challenges of scientific healthcare applications is the streaming of data collected by many sensors deployed across the body. This current situation leads to novel scenarios where there is an intersection between the Internet of Things (with the quantity and quality of new available wearables), Big Data (the enormous amount of data collected by many heterogeneous sources) and real-time environments due to the specific features of the healthcare applications [4]. This new situation is no longer manageable by traditional methods and new computing paradigms need to be defined.

All interconnected sensors create a networked Cyber-Physical System (CPS) [25] that is able to monitor the health of a person [5]. Several strategies have been proposed for specific purposes. For example, the study presented in [26] is a system for long-term monitoring of respiration and pulse. This system consists of four non-contact sensors, used in a laboratory to investigate sensitivity and measuring depth, which are textile-integrated into a shirt. In [27], the authors develop a novel adaptive multi-sensor in order to prevent *venous stasis* condition. This new approach improves and supplements the current therapeutic options by means of pulse synchronous electro-stimulation of the muscle pump.

Recently, different approaches aim to take advantage of the communication capabilities of smart phones in combination with wearable devices to develop versatile monitoring techniques. Some of representative works are described below.

The research work developed by Muaremi et al. [28] shows the use of these devices in the recognition of activities and stages, analysis of group behavior, detection of stressful situations and health monitoring in general. The proposal made by Arsand et al. [29] presents the bidirectional communication between smartphone and smartwatch in order to offer new possibilities in the field of diabetes self-management by providing easier ways of monitoring Blood Glucose (BG), insulin injections, physical activity and dietary information directly from the wrist.

The ubiquitous capability of connected things is being applied in numerous works for remote monitoring of persons at risk, such as the elderly. Terroso et al. [30] present a system consisting of a wearable sensor unit, a smartphone and a website in the senior healthcare field. When the sensor detects a fall, it sends an alert using the smartphone via Bluetooth 4.0 to family members or stakeholders. Yang et al. [31] propose a new method for ECG monitoring based on IoT techniques. ECG data are gathered using a wearable monitoring node and are transmitted directly to the IoT cloud using Wi-Fi. The architecture of the IoT-based ECG monitoring systems is proposed. In this case, all the most complex and computationally-intensive processing is developed in the cloud.

There are techniques developed around smart mobile devices and the cloud [32]. These methods collect environmental information and user data from sensors and use a set of applications to store, process, display, and communicate data to the cloud. Cloud server is the key in processing data. Other works use wearable systems for acquiring physiological data of elderly patients and other environmental parameters of interest [33]. The data is also transferred to cloud applications to be monitored by medical staff.

In all these novel health systems, wearables are able to obtain patient data and send it to smartphones. Smartphones can perform two different tasks: firstly, they send all the data to the server-side layer that processes, analyzes and stores the data, and also shows the processing results; secondly, they are an alternative to processing and analyzing all the data, and presenting the processing results. Table 1 summarizes the contributions.

Moreover, in recent years several approaches over a Mobile Cloud Computing (MCC) environment have appeared in different medical subdomains. The research study developed by Dihn et al. [36] justifies the purpose of applying MCC in medical applications is to minimize the limitations of traditional medical treatment (e.g., small physical storage, security and privacy, and medical errors).

Regarding to the specific case of sport monitoring, several studies have demonstrated the need to monitor the physical effort of athletes to provide knowledge of and tools for ensuring performance maintenance, training programme monitoring, prevention and reduction of athlete overtraining and injury [37,38]. Increasingly, most coaches and training staff have adopted monitoring systems for doing their work more effectively by building custom train and rest plans and preserving the athletes’ health [39,40].

The most basic method for acquiring the information is by means of psychological questionnaires and surveys [41]. The Recovery-Stress Questionnaire for Athletes (RESTQ-Sport) was developed to assess the physical and mental impact of training stress and to facilitate the formulation of strategies for the enhancement of recovery [42]. Other self-report forms such as Profile of Mood States [43] and Daily Analysis of Life Demands [44] are also used. Other reported aspects are the quantity and quality of sleep. The work conducted by Halson [45] demonstrated that sleep loss or deprivation can have significant effects on performance, motivation, perception of effort and cognition as well as numerous other biological functions. Thus, to keep an up-to-date register on sleep quantity and quality can be useful for early detection and intervention before significant performances and health decrements are observed.

The new capabilities of sensors and wearables are able to collect human bio-signals during the training and competition stages establishing an athlete-technology interaction. For example, the research work performed by Jobson et al. [46] is focused on the sport of cycling where power output-measuring devices such as ergometers are used. Another representative example is the study carried out by Twist and Highton [47] focused on the fatigue and recovery aspects of rugby players. Different tests in the team sport context are developed such as the jump test (countermovement/squat jump), strength test, sprint performance, and isokinetic and isoinertial dynamometry as the athlete is doing exercise both during both competition and training time. These studies allow real-time monitoring of the effort rate made by muscles and the measurement of the neuromuscular function. The obtained data includes a number of parameters of physical activity such as average power, normalized power, speed, force, and acceleration.

Another method for monitoring the athletes’ performance consists in the Time-Motion Analysis (TMA). This technique analyses the sequence of movements made by the athletes during the competition using Global Position System (GPS) tracking and advanced movement pattern analysis via digital video [48,49]. The sports intensity may differ according to the playing position and, as a consequence, it has a different impact on the athlete’s stress level and the effort required.

The heart monitoring gives precise information on the physical effort that is taking place at each time. A simple analysis method consists in checking that the heart rate is within a safe range. In this case the system knows that all heart rates for each athlete should be between a lower and an upper threshold. Therefore, if one of the heart rates exceeds the upper threshold or its value is lower than the lower threshold, the system alerts the medical staff. However, other papers on anomaly detection from ECG data are using inference methods based on dynamic programming techniques [50] and other mathematical operations [51] in order to detect deeper heart anomalies that may cause injuries, even when the heart rate is within safe ranges.

The training impulse (TRIMP) is often considered a useful means of assessing training load [52]. A TRIMP is a unit of physical effort that is calculated using training duration and maximal, resting, and average Heart Rate (HR) during the exercise session. There are many research works focused on monitoring of physical activity using HR measures [53,54]. The research work developed by Daanen et al. [55] uses Heart Rate Recovery (HRR) defined as the rate at which HR declines at the cessation of exercise and has been suggested to be a marker of autonomic function and training status in athletes. HRR can be calculated over varying timeframes, usually between 30 s and 2 min, with the difference between end of exercise HR and HR at 60 s post-exercise being most commonly used.

The analysis of the electrocardiographic wave is a powerful method for analysing the performance in sport and detecting heart diseases [56]. There are several risk situations for sport practice that can be inferred from the ECG such as Hypertrophic Cardiomyopathy (HCM) [57], Hypertensive Heart Disease (HHD) [58], commotio cordis [59], coronary artery anomalies (CAA) [60], or left ventricular hypertrophy (LHV) [61].

An important part of the risks associated with physical activity comes from cardiovascular disease and heart failure [62,63], especially, those that can cause the unexpected and SD of the subject. This is known as SD situations in sport. Despite all the recognized health benefits of sport and general physical activity, it has been proven that the practice of sport in an overly intensive way increases the risk of SD. SD occurs in approximately one in 200,000 athletes annually [64]. Recently, the sudden and unexpected deaths suffered by athletes while exercising their respective professional sports have caused some alarm. The phenomenon has been mainly associated with footballers: Antonio Puerta (Sevilla FC, Sevilla, Spain), Marc-Vivien Foe (Yaundé, Cameroon), Miklos Feher (Benfica, Guimarães, Portugal) and Patrick Ekeng (Dinamo Bucharest, Bucharest, Romania). This cause of death is also seen in other professional sports such as basketball, cycling, rugby, volleyball, running, etc. Physical activity is considered a true reflection of the health and quality of life of a society and so the public has trouble understanding how an apparently healthy young person can die while showing great vitality in performing his usual sport. Cases of SD in the headlines of the sports press, especially when it happens to highly-trained athletes with excellent athletic performance, show that the athletes were not aware that they were carriers of a silent yet potentially lethal cardiovascular disease, despite having been subjected to different medical checks throughout their professional sporting lives.

The work presented by Corrado et al. [65] discusses how little is known about the risk of SD in adolescents and young adults engaged in sports. They carried out a study of young people in Italy, with a total population of adolescents and young adults averaged of 1,386,600, of which 112,790 were competitive athletes. They concluded that sport, per se, was not a cause of the enhanced mortality. However, it triggered SD in those athletes who were affected by cardiovascular conditions predisposing to life-threatening ventricular arrhythmias during physical exercise. In the review carried out by [66], a comprehensive assessment of many issues that target the interrelation of intense physical exertion with cardiac structure and function is presented. The less likely potentially adverse consequences of sports are also described. They explore different aspects, such as athlete’s heart, physiology, chamber morphology, spectrum of abnormal ECG, arrhythmias, etc.

Marijon et al.’s [67] study of the prevalence, characteristics, and outcome of sports-related SD represents the first study which assessed and considered 820 cases of sports-related SDs and resuscitated cardiac arrests in a general population. Therefore, taking into account the significant number of cases, the authors identified a higher burden of SD than previously suspected from exhaustive assessments of sports-related SD in young competitive athletes. The objective of the work by Steinvil et al. [68] determined if pre-participation screening of athletes with a strategy including resting and exercise ECG reduces their risk for SD. However, after 24 documented events of SD or cardiac arrest events among competitive athletes during the period between 1985 and 2009, the authors found out that the incidence of SD of athletes is within the range reported by others.

There are other studies investigating SD in general sports in different locations [69,70]. The authors aim to explore sudden cardiac death during physical activity in young adults. Subsequently, these cases were validated by information from medical records and autopsy reports. Twenty-three sports-related SD (17–34 years), were identified [70]. They have even identified the causes of death, which were myocardial infarction, myocarditis, conduction abnormalities, aortic stenosis, cardiac rupture, hypertrophic obstructive cardiomyopathy, congenital coronary anomaly, and coronary sclerosis without defined infarction. Myocardial diseases were the most frequent for those aged under 35 years. These causes of SD could be early detected by comparing the ECG data during sport with the ECG performed during the previous treadmill exercise stress test carried out on the athletes, as demonstrated by the health studies carried out [68].

Other SD methods have been developed in recent times based on the analysis of ECG signal which could be processed in real time and then, to predict SD situations in a preventive way [71,72,73]. This opens new ways of using technology for preventing injuries in sport activities. Moreover, the modern communication capabilities though new protocols allow create a wireless Body Area Network (BAN) of sensors and synchronize the different bio-signals to obtain an integrated profile of the user [74,75,76]. In this way, a wireless sensor network can be implemented and monitoring of a group of subjects. Wireless BAN operates in close vicinity to a human body and supports a variety of applications. IEEE 802 has established a Task Group called IEEE 802.15.6 for the standardization of Wireless BAN [75,77,78].

### 2.2. Findings

After reviewing the representative proposals in this field, some findings can be identified that justify and summarize our contributions to previous works:

- The number of wearables, mobile devices and other connected things are increasing significantly. This increases the possibilities of using new types of applications that take advantage of their ubiquitous sensing and communication possibilities.
- The utilization of user portable things for aiding biomedical sensing is growing. Several works propose using the devices for medical monitoring. In addition, the combination of wearables and mobile devices allow designing biomedical sensing systems for self-monitoring in a comfortable way.
- The complexity of advanced applications means they are expensive to run only on wearables and/or acquisition devices. The trend of upgrading the overall performance of complex applications used in distributed environments (such as IoT, mobile apps, etc.) is finding ways of providing additional computing power to mobile devices and ‘things’.
- The physical effort monitoring in professional sports practice is a very useful tool to understand athletic performance and how the athlete’s body works. The new technology is a key enabler for this purpose.

**Figure 1:**
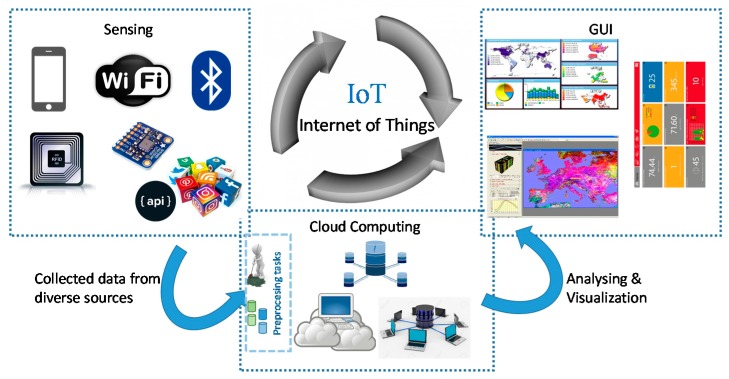
General schema of IoT.

Figure 1 shows a very general IoT scheme, which is the approach shown in most of the works reviewed in the state of the art. There are many tasks throughout the IoT process that can be divided more efficiently.

On the one hand, as far as information input is concerned, IoT establishes a very diverse, distributed, and complex series regarding the great diversity of sensors and other devices/sensing elements that collect data, including social networks through their different APIs. Most of the information is directly sent to the cloud, starting with the previous stages of processing, cleaning, transformation, and normalization, as it has been seen in the works reviewed. However, most of this information can be preprocessed in the available resources on current mobile devices. Likewise, in the last stage of this scheme, analysis, and visualization, again the resources of the mobile devices could play an important role to use their processing capabilities in these tasks.

The research presented in this paper pursues the same objectives as mentioned above and aims to take advantage of the IoT paradigm for designing medical applications and human biomedical monitoring. Our research effort is directed at developing a computational environment integrating heterogeneous computing resources that allows advanced medical application monitoring to be computed. The target devices are those including in the IoT ecosystem around users: wearable devices for measuring different biomedical parameters of athletes in real-time during sporting activities and mobile devices (such as smartphones, tablets, laptops, etc.) where the medical specialist can monitor the athlete performance.

The advantages of the proposed framework are based on the flexibility in distributing the application workload. The capabilities of the processing resources are efficiently used. This fact can be deducted from the described case study and the acquired simulation data such as the formalization capability (of the application and the deployed distributed infrastructure), and the flexibility in distributing the application workload.

## 3. Distributed Computational Framework

### 3.1. Health Monitoring Environments

Modern activities of health monitoring have been revolutionized by development of IoT paradigm. Currently, the new smart sensor devices are changing the way users and professionals can work. In general terms, the combination of sensing technology and smart mobile devices can handle the computing capabilities of new healthcare applications which, not so long ago, had to be performed only in hospital environments.

Several devices, such as smartwatches, mobile phones or tablets have been widely adopted to monitor daily sports activities not only for the athletes but also of the general population, obtaining their activity and performance data [79,80]. In other competitive scenarios, such as professional sports, the medical staff is able to monitor the performance of athletes during times of high stress. For example, in a sporting event such as football match, bicycle or running race. In these cases, the devices usually used are light portable computers like a tablet or laptop. The deployment of powerful workstations or servers are not practical in a real environment. Moreover, the computing aid from a server-side hosted in the cloud is not always available for real-time monitoring. The use of cloud resources is often limited to storing the data for further analysis. These operation conditions limit the type of applications that can be deployed. Thus, nowadays, the most common use by medical staff and sport coaches is just using a monitoring application.

**Figure 2:**
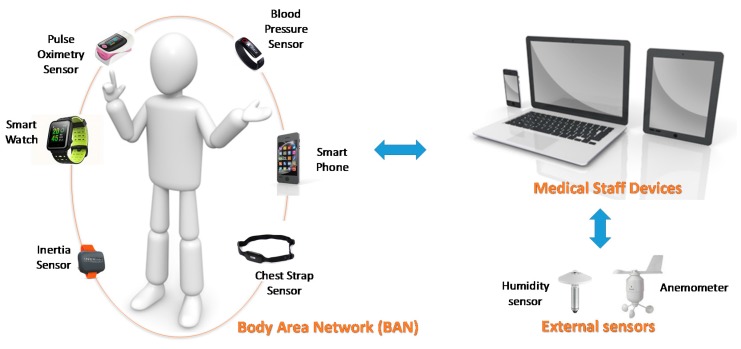
Diagram of the computing elements of the framework.

The proposed framework enhances this health monitoring by means of leveraging the computing capabilities of modern wearables and other IoT devices for computing advanced medical applications. The main idea is to get some devices (sensors or wearables) of the BAN can take part in the application processing and then, to provide high level information to the medical staff’s devices to perform further data analysis. Figure 2 shows the general scheme of the proposed computing elements considered by the framework. In contrast with other approaches [76], the criterion to be a part of this BAN is that the device is worn by the user, and not its processing capability. This approach combines the ‘things’ of the BAN with sensing and computing capabilities in an integrated way. That is, there are thing with only sensing features (i.e., biosensors), there are things with only computing features (i.e., smartphones) and there are things with both sensing and computing features (i.e., smartwatches). All of them have communication capabilities to make a wireless network to share data.

The computing elements are interconnected creating the IoT communication network. Usually, the set of devices are connected to a Local Area Network (LAN) in a wireless way. Thus, these devices form a wireless local area network (WLAN) among them. The development of the WLAN standards has been driven for bringing connectivity anywhere and improving the performance of the network. Table 2 summarizes some features relating to transfer rate of the latest evolutions of the standard up today [81,82].

The maximum number of supported devices or stations connected to a WLAN depends on the data rate of the connection since they have to share the available bandwidth. The current transfer rate can be known for each device by simply monitoring the network interface. However, in order to get a relative value, the total bandwidth available for the device is required. In a network where computing nodes are mobile and connected through standard wireless networks, knowing the total free bandwidth is not an easy task. First of all, in standard networks, bandwidth is shared among the interconnected devices, and secondly, the position of the mobile devices affects the available bandwidth. In such cases, one feasible approach is to consider an estimation of the total bandwidth based on the average values retrieved on regular checks by the devices.

### 3.2. Framework Specification

The framework provides a method to successfully design and configure the distribution and sharing of the application workload. Table 3 gives a general overview of the inputs and outputs for each design stage. As can be appreciated, the design of distributed applications under the proposed framework consists of three main steps: (i) application analysis for task and data flow break down; (ii) resource planning and (iii) deployment and empirical adjustments.

Next, explanations on how the framework works are provided. The example used corresponds with the SD detection in mobile environments. This methodology can be exported to other advanced healthcare monitoring applications or other contexts, especially those where intensive data acquisition and high processing needs take place.

The applications based on ECG analysis, and in particular, the SD detection have been reviewed in the state-of-the-art section to show the importance of providing solutions able to be deployed in mobile environments where a hospital monitoring context is not available.

(i) Application analysis for task and data flow decomposition

There are different ways in which the overall activity of the application can be decomposed in different tasks. This work depends on the application area and the type of application. There are two key features in this step.

Firstly, a proper application partitioning to distribute the code for local and remote computation must be designed. The methodology can be static or dynamic: the static approach establishes the parts of the candidate application to be processed by smart sensors at the design stage; the dynamic approach requires analyzing the application code on the fly to determine which part can be processed out of the device.

Secondly, the granularity unit needs to be defined. This feature determines the size unit of the application that can be computed separately. The usual options are module, process, class, component and method. This granularity is used for partitioning the application. This aspect is closely related to the abstraction level and data transmission requirements. A coarse-grained allows a high level of abstraction but increases the need for communicating application details and synchronization. In contrast, a fine-grained needs so much scheduling work. This framework considers as granularity unit the application task.

The review of existing implementations and state-of-the-art techniques are very important for designing the right decomposition of the applications. In addition, the application requirements and the working environment constraints play an important role in designing the proper data-flow diagrams. Generally, modern implementations of many computer methods take into account limited-resource target devices such as wearables and sensing devices. In these cases, some key features must be considered such as the enhanced communication capabilities and battery consumption.

As a result, the target application is described by a directed graph 𝔸 = {𝕋, 𝔽} where:

- 𝕋 is the vertex set and represents the set of application’s tasks required for data acquisition, processing and monitoring. Then, the application is broken down into a list of tasks: 𝕋 = {t_1_, t_2_, …, t_n_}
- 𝔽 is the edge set and represents the data flows exchanged between the tasks. The data flows set the precedence between the tasks and the volume of exchanged data. F(i, j) ∈ 𝔽 defines the volume of data exchanged between the tasks i and j.

For the SD application example conducted along this section, a lot of work have been reviewed in this research in order to design the proper data flow decomposition. Thus, the list of identified tasks corresponds with the most common processes involved in the SD detection mentioned in the state-of-the-art techniques.

**Figure 3:**
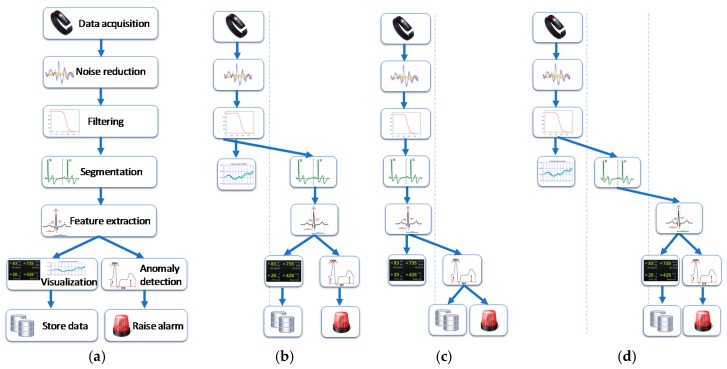
Diagram of a SD detection application in our proposed IoT-based framework. (**a**) List of tasks and general flow; (**b**–**d**) Distributed Configurations.

The data flow diagram shown in Figure 3a depicts an example of an application modelled according to this principle. This task size can be one of the previously mentioned, it can be variable-sized and executed sequentially.

(ii) Resource planning

Once the computational load is characterized, the next step consists of determining where each task will be processed. The idea of the framework is that the processing flows from BAN devices to other devices outside it. In this step, the network architecture, and the IoT environment are essential inputs for carrying out the resource planning.

In accordance with the proposed framework, the devices ⅅ involved are defined as follows:

- Let B be the set of devices or ‘things’ of the BAN. These devices are worn by users. Their work consists in sensing and communicating the data to other devices. In addition, they may have some computing power.
- Let M be the set of available computers outside the BAN of the user. This set includes the computers and other mobile devices that have processing capabilities. Thus, the devices of this set can show the data and process it.
- Let E be a set of external sensors. This set includes another type of external devices to the user for sensing other information for the application. For example, environmental conditions such as ambient humidity and temperature.

That is: ⅅ = {B} ⋃ {M} ⋃ {E}

In this way, the computation begins inside the BAN where the data are acquired and can be complete in other more powerful devices, that is from B to M devices. Although possible, a data stream back is not contemplated from the M to the B devices. The ideal configurations are expected to distribute incrementally the processing of the application with the objective of computing the raw acquired bio-signals in the nearest location for the user, and reducing the communication needs with the M devices.

This causes two main positive effects: on the one hand, the user can monitor elaborated data, and, on the other hand, the medical devices increase their capability of handle more users at the same time. This second advantage comes into play especially with complex applications and where there are many users to be monitored, for example, the number of players of a football team is eleven, in a running race there could be hundreds of athletes. As a result, the proposed framework creates an intelligent BAN to enhance the processing of advanced healthcare applications and could improve the quality of medical monitoring and health analysis.

This approach transforms the application into a distributed application represented by the directed graph 𝔸, and which can be processed along the set of devices ⅅ. For example, Figure 3b–d illustrate some configurations of distributed computation. Rather than just send the data to the medical staff computers, these scenarios allow a distributed processing of the application. In the case Figure 3b, the first three tasks can be performed by the devices within the BAN and show some data which can be obtained directly from the acquired data to the user, for example, heartbeat count and its evolution along the time in a graph. In the case Figure 3c, further processing is performed to obtain elaborated and precise information before sending them for anomaly detection. The configuration Figure 3d depicts a scheme where two external devices to the BAN come into play: the computation begins inside the BAN, a middle device performs a single task and the rest of the work is processing in other device. Other scenarios can occur depending on the characteristics of the deployed infrastructure and the application decomposition.

(iii) Deployment and calibration of the system

In the distributed system, each task can be run on a different device with different capabilities. To establish the most suitable computer where to run each task, a set of factors must be taken into account: computing resources, battery consumption, network bandwidth availability and latency, etc. In addition, most of these factors could vary over time; for example, the free network bandwidth.

For each computer or computer profile, the resources defined in the previous section need to be configured. After that, the performance of the key devices under each profile and context application should be carefully measured. It is necessary to check that the device has always enough capabilities for meeting the requirements. In addition, the distributed application must offer results as expected, switching data flows between devices according to defined data-flow and distributed configurations. The resource planning step should be reviewed and adjusted until the overall system works as expected; proper system modelling and simulation can help in the successful completion of this step.

## 4. Case Study

In this section, we present a case study in order to validate our proposal and shown the benefits of the framework in providing flexibility for sharing the processing of advanced health monitoring applications. The case study describes some working scenarios and offers several implementation alternatives depending on the aims of the acquired data and the available computing resources.

### 4.1. Case Description

An increasing number of coaches and individual athletes are becoming more and more convinced of the benefits of such wearable bio-sensors to obtain valuable indicators. The smart systems created around them allow sports performance to be improved, monitoring individual effort, and preventing injuries or even SD. Nowadays, wearing a chest strap during sports is very realistic, especially in top-level sports where athletes need to collect data constantly to compare them with historical registers. In addition, the technological development of sensing technology will allow to create advanced body devices working in an “unnoticed” mode without rigid parts (as smart clothes, patch or skin sensors) and where wires were not necessary and able to overcome the interferences from motion artifacts [83,84,85].

This proposal consists of monitoring the heart information of each player during a football match. The idea of football game is due to the media transcendence, but this idea can be extended to any other sport where a variable number of players takes place in a mobile environment. For example, running, cycling, etc. The healthcare application consists in monitoring the ECG of each athlete in order to prevent the sudden cardiac death during the match.

The process is based on continuously analyzing the information retrieved from the “standard 12-lead ECG”. This data provides specific information from the different areas of the heart. This information is usually used for diagnosis in electrocardiography issues [56,86,87] and it can be read by a chest strap wearable sensor. By means of this monitoring and analysis process, the system can infer the main ECG-based Key Performance Indicators (KPIs) for predicting a heart attack in a player.

In the mentioned application scenario, the proposed framework can provide processing distribution by using the methodology depicted in Table 3. First, the overall activity must be properly broken down into tasks and data flows. As a simple example of the functions that take place in ECGs analysis [88,89,90,91,92,93,94,95,96], the following tasks are defined according the scheme depicted in Figure 3a. Other tasks and operation may be needed for each of the previous diagnosis methods.

Tasks ≡ 𝕋 = {t_1_: data acquisition; t_2_: noise reduction; t_3_: filtering; t_4_: Segmentation; t_5_: Feature extraction; t_6_: anomaly detection; t_7_: visualization; t_8_: Store Data; t_9_: Raise alarm}.

t_1_: *Data acquisition*: The data acquisition is always made by the biosensors. A modern ECG device is basically a digital system. Before any processing, the analog ECG signal is immediately converted into a digital form by an Analog to Digital Conversion (A/D) at a particular sampling rate or frequency for further usage.

t_2_: *Noise reduction*: The noise reduction is an essential task for ECG analysis. The bio-signals can be extremely noisy due to the extremely weak signals read. It is expected that this task only removes the noise without changing the desired signal [88]. Thus, this task only cuts off low and high frequency noise.

t_3_: *Filtering*: This task is generally designed to attenuate or remove some frequencies from the input data, enhance signal and make the work of the next tasks easier. There exist several types of methods for filtering a signal: linear, nonlinear and polynomial filtering, high-pass filters, finite impulse response filters, etc. [89].

t_4_: *Segmentation*: Heartbeat segmentation allows to analyze the ECG signal. Different methods have been used to improve the detection accuracy of QRS complex [96]. For example, the Hilbert transform allows to detect the R peak and to segment the morphology of QRS complex along the ECG [95]. A wide range of methods allowing high detection rates have been proposed, and recently, there are research works for achieving a real-time detection process in order to run this ECG analysis applications into wearable biosensors [90,91,92,93].

t_5_: *Feature extraction*: This is the core tasks of the ECG analysis. Any information extracted from the heartbeat used to discriminate its type maybe considered as a feature. Using the wavelet transforms is a suitable method for extracting features from the ECG signal from both frequency and time domains [97,98].

t_6_: *Anomaly detection*: this task consists in inferring the corresponding user’s heart condition: healthy, heart failure, myocardial infarction, etc. In this field, there also exists intensive research work [50,51]. Recently, machine learning methods offer a good tradeoff between accuracy and computational cost [94,99].

t_7_: *Visualization*: Visualization of the data and results. This task can show the information in real-time or on-demand. The medical staff can obtain aggregate data from the team of players.

t_8_: *Store Data*: The data and the results of analysis can be stored in the computer for further processing. For example, conducting aggregate big data analysis.

t_9_: *Raise alarm*: In case where the user is in a risk situation for health, the system can raise an alarm to the medical staff and to the user itself.

The relations between the tasks are defined according to the data flows depicted in the figure. Some tasks can be processed sequentially and others can be performed in parallel. The results obtained can be stored, displayed on the mobile device and/or an alarm can be raised if necessary.

### 4.2. Infrastructure Deployment

The next design step is to set the devices that will collaborate in sensing the data and processing each task. This is the set of the devices ⅅ, and it may contain local sensors, wearables, smartphones and tablets. These devices are in the IoT communications network. The set of devices of the BAN consists of a chest strap sensor and a smartwatch.

- The chest strap sensor is a wearable biomedical sensor that reads athletes’ heart rates. Each athlete should wear this sensor for acquiring the most relevant data. The device basically consists of a chest strap sensor that monitors the heart rate and a communication module for sending the data to the display devices. This device does not have computing capabilities due to size and battery constraints. Thus, the chest strap sensor is limited to sensing the heart rate per second and sending it to the athlete’s smartwatch. A Bluetooth link is established to accomplish this goal.
- The smartwatch is a modern wearable basically designed for display purposes. The device can acquire other data such as the field position, the distance covered, GPS and altitude position and the number of minutes played. The wearable has good communication features. Usually, it supports several communication standards such as Bluetooth, WLAN and Global System for Mobile Communications (GSM) modes. In our proposal, each smartwatch receives the athlete’s heart rate from the chest strap sensor and makes a data package that includes the heart rate, GPS coordinates (position in the field), covered distance and number of minutes played. This data package is sent to the mobile devices of the medical staff repeating this process each second. The communication between them is by means of a dedicated WLAN network.

In addition, an environmental sensor has been added to the system. The ambient conditions could influence the performance of the athletes and the risk of SD. In this case, the IoT environment can take into account more than just the health sensors in order to reach a better prediction. The environmental sensor reads ambient conditions during the match such as ground temperature, wind speed, wind temperature, and ambient humidity. These data are packaged and sent to the mobile devices of the medical staff repeating this process each minute.

**Figure 4:**
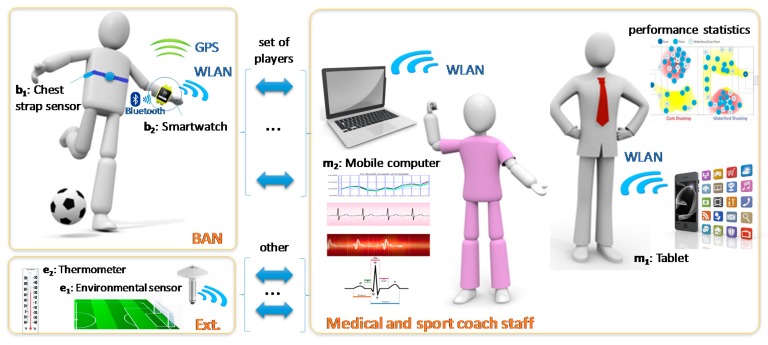
Application scheme.

The available computing devices are of different types. Firstly, the athletes’ smartwatches can perform some processing work. In addition, the sport coaches and medical staff have mobile devices (such as smartphones, tablets or laptops) for performance assessment and medically monitoring the players. Figure 4 shows the scheme of the infrastructure deployment, the communication methods and the users of the system.

According to this deployed infrastructure, the IoT environment consists of the following devices (Figure 4):

B = {b_1_: chest strap sensor; b_2_: smartwatch}
M = {m_1_: coach’s tablet; m_2_: medical staff’s mobile computer}
E = {e_1_: environmental sensor; e_2_: thermometer}

So that, ⅅ = {b_1_, b_2_, m_1_, m_2_, e_1_, e_2_}

The IoT communication network uses different modes. Communication between the biosensor that footballers wear on their bodies and the smartwatch is made through Bluetooth. This method is usually used for exchanging data over short distances using short-wavelength Ultra High Frequency (UHF) radio waves [100]. The communication between the wearable and the remote devices is performed by means of a dedicated WLAN deployed on the football pitch. The next generation of 802.11ah standard is being developed for IoT applications. Its main features are greater coverage and higher power efficiency [81,82]. This standard can cover a long distance for outside environments that is sufficient for a football match. Finally, the environmental sensor data are communicated via wired LAN to be displayed and/or processed.

The medical staff has a set of mobile devices that receive all the data packages from the athletes’ smartwatches and the environmental sensors. The raw data are locally stored in an SQLite database. Finally, when the match finishes, this local SQLite database can be sent to the cloud server to build historical user database for further processing and the development of more exhaustive cardiac checks for them.

Other scenarios could be designed according to the infrastructure availability and the application’s objectives. For example, the coach staff of each team is able to monitor the data and obtain performance statistics for sport-related reasons. That is, monitoring whether an athlete is doing as well as they should be.

### 4.3. Distributed Processing

For many amateur sports, the objective of the system is only to present the information to the user and create a database with historical data. But for professional athletes for whom greater physical exertion is required, other more elaborate calculations are needed.

The development of mobile computing has enabled to compute complex applications and methods in mobile devices. This is the case of portable Personal Computers (PC) or laptops, tablets or even modern smartphones. The latter has experienced a spectacular computer power increase to face the demanding requirements of new commercial Apps [101]. In addition, the ubiquitous nature for computing and sensing make them suitable devices for some healthcare applications.

Some of the techniques and methods reviewed on the state-of-the-art section could be processed on smartphones [102,103,104]. However, there are important shortcomings for computing advanced healthcare applications on mobile devices. In first place, the battery life of these devices is an important issue and it does not allow a high intensity processing for a long time. In addition, the smartphone or other mobile devices are too heavy and sized to be worn by athletes in professional sports. Secondly, although the medical staff or coach team can handle this kind of devices for health monitoring, the power performance is not enough for simultaneously monitoring a numerous group of athletes. In addition, for some scenarios, the deployment of powerful workstations or accessing to external cloud computing resources are not available options.

In the proposed case study, the biomedical data needs to be processed as quickly as possible to obtain a result that allows medical staff to react rapidly. Moreover, the health information obtained could also be used for performance assessment of the sport activity. Three working scenarios are described to show the flexibility of the framework in handling variable workload and distributing the processing.

- Application context A consists of a single athlete in a training session.
- Application context B consists of a training session of a group of 11 athletes. In this case, all footballers must be analyzed at the same time.
- Application context C represents a football match where there are two teams of 11 athletes and four referees to monitor. That is 26 athletes in total.

Due to the different and exhaustive medical checks that are performed on footballer’s hearts in addition to the treadmill exercise stress tests, all the good and bad heart rates should be known for each of the players and the referees. This volume of data could outperform the computing capabilities of the mobile devices for processing the whole application.

The application tasks shown in Figure 3 need to be processed on the fly to provide the right information on time. For early SD prevention, a few seconds is needed by the cardiologist team to make the right decision and save the footballer’s life. The system provides medical staff with more elaborate data and alerts in high risk situations.

The proposed framework can overcome these drawbacks by providing a distributed computation approach. However, the decision on what tasks may be computed on the BAN’s devices is not trivial. Wearable devices, such as smartwatches, do not have enough computing power for performing complex calculations. Their contribution in the processing will not be great, but sufficient for helping in providing the results on time.

The idea of sharing the processing makes that part of each algorithm were processed by each athlete at the same time. Note that this is not a scaling problem, instead, flexibility is needed. The capability for handling the complex IoT scenarios provided by the proposed system is independent of the amount of data to be processed at the same time. For example, a marathon in which thousands of runners participate could be monitored in a similar way.

The framework enables the task distribution and processing through computing-resources shared among available devices of the mobile environment. To configure the behavior of the system and the correct distribution of the tasks, the computing load must be defined in terms of the performance aspects for each t_k_: response time and transfer rate for each device. The most suitable devices can be set up for each application depending on the performance aspects and costs estimations.

This case study considers the distribution of tasks 𝕋 depicted by Figure 3b–d. These are only possible scenarios. Other distributions can be configured according to the complexity of the methods and the healthcare application, the available devices of the BAN and the amount of computing resources outside of the BAN.

The suitable task distribution can be done after a calibration process of each scenario. The computational framework sets up the tasks that will be processed on each device and defines their efficient distribution according to the application needs, the available resources and the user preferences. The data flow switching between sensors and devices under the established task schedule gives an overall resilience for the mobile environment and allows the healthcare application meets its requirements.

As shown in the figure, data acquisition (t_1_: data acquisition) is always made through devices of BAN. The preprocessing stages (t_2_: Noise reduction; t_3_: Filtering) are serious candidate to be processed on the sensing devices and/or other devices of the BAN. In this way, the BAN supplies clean data instead of raw data. The next operations (t_4_: Segmentation; t_5_: Feature extraction; t_6_: anomaly detection) need some computing power to be performed. If it is possible to be computed inside the BAN, it provides health information rather than a data signal. In addition, it may reduce the amount of data to be transmitted and the bandwidth is most efficiently used.

There are recent research work aiming to reduce the computational cost of the ECG analysing techniques [105,106] for enabling their processing in wearable devices. This is the scheme followed by Figure 3c.

Finally, there exist tasks that could be done by several devices at the same time. For example, the data can be *visualized* by the player’s wearable, the medical staff’s tablet and the mobile computer in order to inform all potential beneficiaries of the data.

### 4.4. Results and Discussion

**Figure 5:**
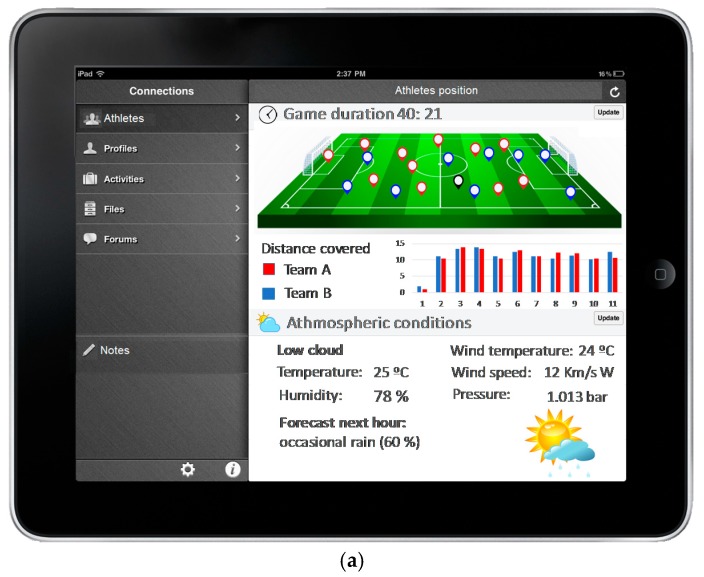

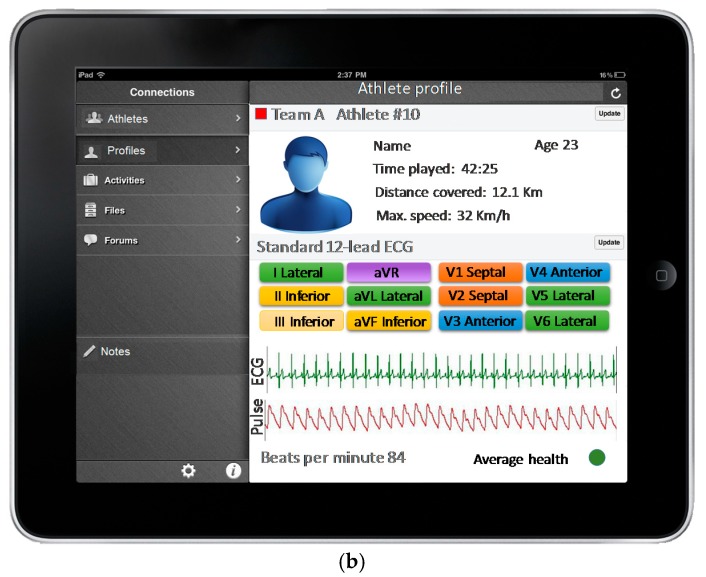
Scheme of the monitoring application. (**a**) Overall environment conditions (**b**) Mockup of ECG parameters of football players.

Figure 5 shows some mockup applications and a scheme of the information provided by the proposed system in performing advanced monitoring and analysis applications. The candidate parameters to be analyzed for each player and for each football match are cardiac parameters, number of minutes played during the match, ambient temperature, ambient humidity, altitude and distance covered by the player. All these parameters serve to measure the degree of effort of each player during the football matches and the players’ physical exertion.

The experiments conducted have been designed for the application described. The design of the test is based on the results of previous research works [88,93,103,104] and on our own estimations. In addition, recent methods that propose a real-time segmentation and feature extraction have been reviewed in order to get an improved implementation for wearable and mobile devices [90,91,92,93]. The validation of these methods is usually produced in term of *Sensitivity* in detecting anomaly behaviours. However, enhancing the detection accuracy is not the main focus of this work. Rather, we strive to provide a flexible approach for leveraging the capabilities of modern sensing and wearable devices in computing complex applications.

Table 4 shows the estimation delay cost for the tasks described above. This performance depends on the input data sizes. In this work, we consider 1 KB/s the size of data acquired from a “standard 12-lead ECG” sensor device. The computing platforms are (b_2_) a smartwatch equipped with an ARM A7 at 1.4 GHz processor, a (m_1_) tablet with Qualcomm Snapdragon 820 CPU at 2.15 GHz, and a (m_2_) portable computer with an Intel(R) Core(TM) i7-7660U CPU at 2.50 GHz processor. The performance ratio between these devices for a constant input data size according their specifications is estimated in: 1× for smartwatch, 3.93× for tablet device and 4.56× for the laptop. That is, in average, the m_1_ is 3.93 times faster, and the m_2_ is 4.56 times faster than the b_2_ device, respectively. In addition, the portable computer can be equipped with specialized hardware to enhance the digital signal processing, for example, the computation of wavelet transform of the methods involved in computing the t_5_ task.

It is assumed that highest possible heart rate is around 220 beats per minute, including under condition of intensive sport activity. Thus, after a pulse is detected, it is physiologically impossible for another beat to occur before 0.27 s. This sets a time constraint of 0.27 s for anomaly detection. This is the time constraint of the application for working in real-time situations. This information along with the data shown in the previous table are the input of the computational framework. The design of the data-flows depicted by Figure 3 is the result of the calibration of the system step of the framework, after simulating the delay cost of the tasks in each device for each application context. Other input information can drive to other suitable configurations for distributing the application.

To properly computing in real-time the healthcare application in the different application contexts, the workload is distributed among the available resources around the mobile environment. Unlike other proposals for conducting the ECG analysis offline [72,73] or inside the wearables [102,103,104], or even in the cloud [31], this approach allows leveraging the available infrastructure and perform the healthcare monitoring on the fly for a variable number of users.

Table 5 shows the computing cost of the whole application according the configured scenarios depicted by Figure 3 for each application context. This data represents the flexibility of the framework to handle an increasing workload by distributing the tasks and using different computing platforms.

The previous tables allow to draw some conclusions on how the framework opens computing possibilities to handle monitoring applications in different operation contexts and verify the variable use of resources in mobile environments. For training monitoring of a single athlete (application context (A)), as shown in Table 4, all the devices (b_2_, m_1_ and m_2_) can provide a real-time processing of the healthcare application. It is the classic scheme for self-monitoring. In a training session where a group of athletes must be monitored for healthcare and/or performance in sport purposes (application context (B)), the wearable devices of the BAN cannot provide all information since the data needs to be centralized and be compared with information from databases. In this case, the computation is distributed using a combination of devices of BAN and devices of medical staff and/or sport coach, for example the tablet device. In this case, as shown in Table 5, the configurations Figure 3c,d can provide the results in time for all the players. For Figure 3c, with just the m_1_ device the data from all athletes can be provided. Finally, in a football match with 26 on-field athletes (application context (C)), the m_1_ device is not sufficient to give enough computing power and the only configuration to supply the data on time is Figure 3c.

Other data sources and computing devices could produce different performance results, and, of course, more hardware resources can be deployed in order to provide more computing power. However, the main idea is to make the best of the potential of *things* for healthcare monitoring and distributing the processing among the available surrounding devices. Not only in sensing, but also, in computing the health applications. Thus, the involvement of the BAN devices enhances processing performance and reduces the need for external hardware, thereby leveraging the network resources more effectively.

The flexibility provided by the system allows it to be run on heterogeneous IoT environments where the numbers of users and devices are changing. For single users, personal trainers and amateurs, the main tasks of the application can be computed both by external resources and mobile things. The described case application is a simple illustrative example where the infrastructure can be insufficient for providing the necessary computing power. In other scenarios, with thousands of users (i.e., a marathon) dedicated hardware platforms are insufficient for processing the data. In these cases, the presented proposal provides a solution for drastically reducing the hardware requirements and solving complex biomedical applications across many users by using all available resources.

## 5. Conclusions and Future Work

The computing requirements for monitoring and advanced analysis of the data acquired by IoT environments can overcome the capabilities of the sensors and personal computers deployed by specialists. Additional specialized hardware is usually required to meet the application requirements. In this work, a distributed framework that combines sensing and processing at different levels of the network to share the computing load among the available devices has been proposed to address this challenge. The IoT environments composed of wearables and other biosensors may benefit of it by allowing the processing of advanced applications with real-time constraints in a collaborative way.

The main advantages and novelties of the proposed system is the flexibility in the application execution by using resources from different available devices. In this way, the devices of BAN can provide shared computing resources that enables real-time monitoring and analysis of all acquired data.

The health monitoring and SD detection in sport is a good scenario where the proposed system demonstrates its capacity for handling intensive data processing applications. In this case study, the real-time needs for cardiac information of athletes can be solved by applying a real-time heart telemetry system capable of collecting the main cardiac parameters. Then, our approach broadcasting alerts when any of those parameters exhibit different behavior compared to normal cardiac activity in situations that require athletes to perform under physical effort. In those situations where cardiac disorders exist and the cardiologist team has only a few seconds to make the right decisions to provide the best medical care for the athlete, our system provides them with precise information for doing that.

This proposal provides two main research contributions: a strategy to optimize the use of biomedical sensors and computing resources to deliver advanced applications to the user, and the design of a distributed computational framework that uses the available computing capabilities in the smart devices for sharing the whole processing of the health monitoring applications.

The framework will need to be adjusted when used in real situations. It appears to work in sports but it would be necessary to test this in a real situation. The technology and formalization of the domain knowledge are the limitations and we need to do an in-depth study of these issues for future work. To this end, we have already established contact with and had several meetings with the directors of sport centres and gyms who are interested in evaluating of our framework.

This proposal can be applied to other IoT scenarios, especially those where intensive data acquisition and high processing needs take place.

Further work must be invested in building a proper predictive model and exploit the off-line possibilities of processing the information gathered. In this way, interesting issues come from analyzing athletes’ achievements, movements and each other interactions. This could be made by computing the differences between current and stored athlete profile, as well as making the classifier that detects dangerous patterns.

## Figures and Tables

**Table 1:** Recent biomedical sensing research works.

Research	Biomedical Signals	Devices
Real-time streaming data in healthcare applications [34]	Generic Biomedical signals	Generic Biomedical sensors
Recognition of activities and health monitoring [28]	Heart biomedical signals	Smartphones & wearable devices
Long-term monitoring of respiration and pulse [26]	Respiration and pulse	Non-contact sensors textile-integrated
Diabetes monitoring [29]	Daily activity data	Smartphone & smartwatch
Active assistance [30]	Activity and environment data	Wearable sensors and smartphone
Detect and prevent venous stasis [27]	Pulse and blood flow data	Multi-sensor plethysmography device
Physiological data of elderly patients [33]	Oxygen saturation level, Heart Rate	Biomedical sensors & smartphone
ECG Smart Healthcare monitoring [31]	ECG signals	Wearable ECG sensors and Cloud for processing
Mobile medical computing systems [32]	Medical signal and context information	Different sensors and actuators
Applications in the pervasive environment [35]	Pulse rate, blood pressure, level of alcohol, etc.	Mobile healthcare

**Table 2:** Current WLAN standards features.

Technology (Release Date)	Frequency Band	Data Rate *	Range *	Target Applications
802.11n (2009)	2.4; 5.4 GHz	600 Kbps	30 m	Standard scenarios.
802.11ac (2014)	5.4 GHz	1.3 Mbps	30 m	High speed scenarios (i.e., home, hotels, airports, etc.)
801.11ad (2012)	60 GHz	7 Gbps	10 m	High density and/or extra-high speed indoor scenarios (i.e., conference room, department).
802.11ah (2016)	0.9 GHz	100 Kbps	1000 m	Indoor/outdoor IoT scenarios.

* The data depend on the installation scenario and environmental conditions: i.e., indoor, outdoor, presence of walls and obstacles, etc.

**Table 3:** Framework methodology.

Design Stages	Inputs	Outputs
(i) Application analysis for tasks and dataflows break down	Implementations State_of_the_art techniques Application requirements Working environment constraints	Application partitioning Granularity unit Data-flow diagrams
(ii) Resource planning	Cloud market Network architecture IoT environment	IoT environment configuration: sensors, wearables, mobile devices, etc.
(iii) Deployment and calibration of the system	Configuration set up. Test	Distributed architecture for IoT environment

**Table 4:** Time estimation for SD detection in application context (A).

Task	Smartwatch (b_2_)	Tablet (m_1_)	Portable Computer (m_2_)
*t_2_*	0.0000228	0.0000058	0.0000050
*t_3_*	0.0001824	0.0000464	0.0000400
*t_4_*	0.0200000	0.0050891	0.0043860
*t_5_*	0.1800000	0.0458015	0.0026316
*t_6_*	0,0223440	0.0056855	0.0049000
Total	0.2225492	0.0566283	0.0119625

Units in seconds (s).

**Table 5:** Time estimation for SD detection in application contexts (B) and (C).

	Application Context (B) − 11 Players			Application Context (C) − 22 Players + 4 Referees		
Task	*Figure 3b*	*Figure 3c*	*Figure 3d*	Figure 3b *	Figure 3c *	*Figure 3d*
*t*_2_	0.0000228	0.0000228	0.0000228	0.0000228	0.0000228	0.0000228
*t*_3_	0.0001824	0.0001824	0.0001824	0.0001824	0.0001824	0.0001824
*t*_4_	0.0559796	0.0200000	0.0559796	0.1140351	0.0200000	0.0735235
*t*_5_	0.5038168	0.1800000	0.0289474	0.0684211	0.1800000	0.0684211
*t*_6_	0.0625405	0.0625405	0.0539000	0.1274000	0.1274000	0.1274000
Total	0.6225421	**0.2627457**	**0.1390322**	0.3100613	0.3276052	**0.2695498**

* The computing is outsourced to the portable device; Units in seconds (s).
